# First description of the performance of the VITEK MITUBE device used with MALDI-TOF mass spectrometry to achieve identification of gram-negative bacteria directly from positive blood culture broth

**DOI:** 10.1128/jcm.00122-25

**Published:** 2025-07-18

**Authors:** Daniel D. Rhoads, Nancy D. Hanson, Kaley Reedy, Johanne Gafsi, Yun X. Ying, Dwight J. Hardy

**Affiliations:** 1 The Department of Pathology & Laboratory Medicine, Cleveland Clinic 685191 , Cleveland, Ohio, USA; 2 The Department of Pathology, Cleveland Clinic Lerner College of Medicine, Case Western University 161821, Cleveland, Ohio, USA; 3 The Infection Biology Program, Lerner Research Institute 22516 , Cleveland, Ohio, USA; 4 Department of Medical Microbiology and Immunology, Creighton University School of Medicine 12282, Omaha, Nebraska, USA; 5 Creighton Center for Antimicrobial Resistance and Epidemiology, Creighton University School of Medicine 12282, Omaha, Nebraska, USA; 6 bioMerieux Inc. 355122 , Hazelwood, Missouri, USA; 7 bioMérieux, SA 1896, Marcy-l'Étoile, France; 8 The Department of Infectious Disease, Quest Diagnostics at Medfusion, Lewisville, Texas, USA; 9 Clinical Microbiology Laboratories, UR Medicine Labs, Pathology and Laboratory Medicine, University of Rochester Medical Center 6923, Rochester, New York, USA; Endeavor Health, Evanston, Illinois, USA

**Keywords:** MALDI-TOF, mass spectrometry, rapid tests, blood culture

## Abstract

Improving the rapidity of identification of bacteria causing bacteremia continues to be a focus for quality improvement within the discipline of clinical microbiology. This multicenter study was used to obtain In Vitro Diagnostic Regulation Conformite Europeenne (CE) mark in Europe and describes the performance of the VITEK MITUBE to matrix-assisted laser desorption/ionization (MALDI) workflow for identification of 10 species of gram-negative bacteria directly from positive blood culture broth without subculture. One hundred twenty-five (125) prospective clinical samples, including *Escherichia coli* (69), *Klebsiella aerogenes* (4), *Klebsiella oxytoca* (3), *Klebsiella pneumoniae* (29), *Proteus mirabilis* (9), *Pseudomonas aeruginosa* (10), and *Serratia marcescens* (1), were included; and 123 (98.4%) were accurately identified using the VITEK MITUBE workflow. None were misidentified, and two (1.6%) *K. pneumoniae* were not identified. Contrived samples were also tested and reported in this study. When considering both the prospective clinical samples and the contrived samples, the workflow was able to identify the species 95% of the time. *Proteus vulgaris* accounted for 81% (13/16) of the samples that were unable to achieve an identification. A single misidentification error is reported in the contrived sample testing. The VITEK MITUBE to MALDI workflow enables accurate and reliable identification of commonly encountered gram-negative bacteria directly from positive blood culture broth.

**IMPORTANCE:**

MITUBE is a new *in vitro* diagnostic device designed to meet a need in the clinical microbiology community for a simple, rapid, accurate, and inexpensive system to identify bacteria detected in blood cultures using MALDI-TOF and without the need for subculture.

## INTRODUCTION

Rapid detection and characterization of bacteremia continues to be an important focus of the clinical microbiology laboratory. Marketed technologies that are designed to clean up microbes from a positive blood culture (PBC) include Accelerate Arc, Bruker SepsiTyper (1), Selux PBC Separator (2), and formerly Qvella’s Liquid Colony system (3), and this study describes the VITEK MITUBE (bioMerieux) device, which is also designed to prepare a PBC for matrix-assisted laser desorption/ionization time-of-flight mass spectrometry (MALDI-TOF MS). VITEK MITUBE has received In Vitro Diagnostic Regulation (IVDR) status in Europe, but it is not marketed in the U.S.

This multicenter study used for the CE mark under European Medical Device In Vitro Diagnostic Regulation 2017/746 (IVDR) describes the performance of the VITEK MITUBE to MALDI workflow for identification of 10 species of gram-negative bacteria directly from PBC without subculture.

## MATERIALS AND METHODS

**Fig 1:**
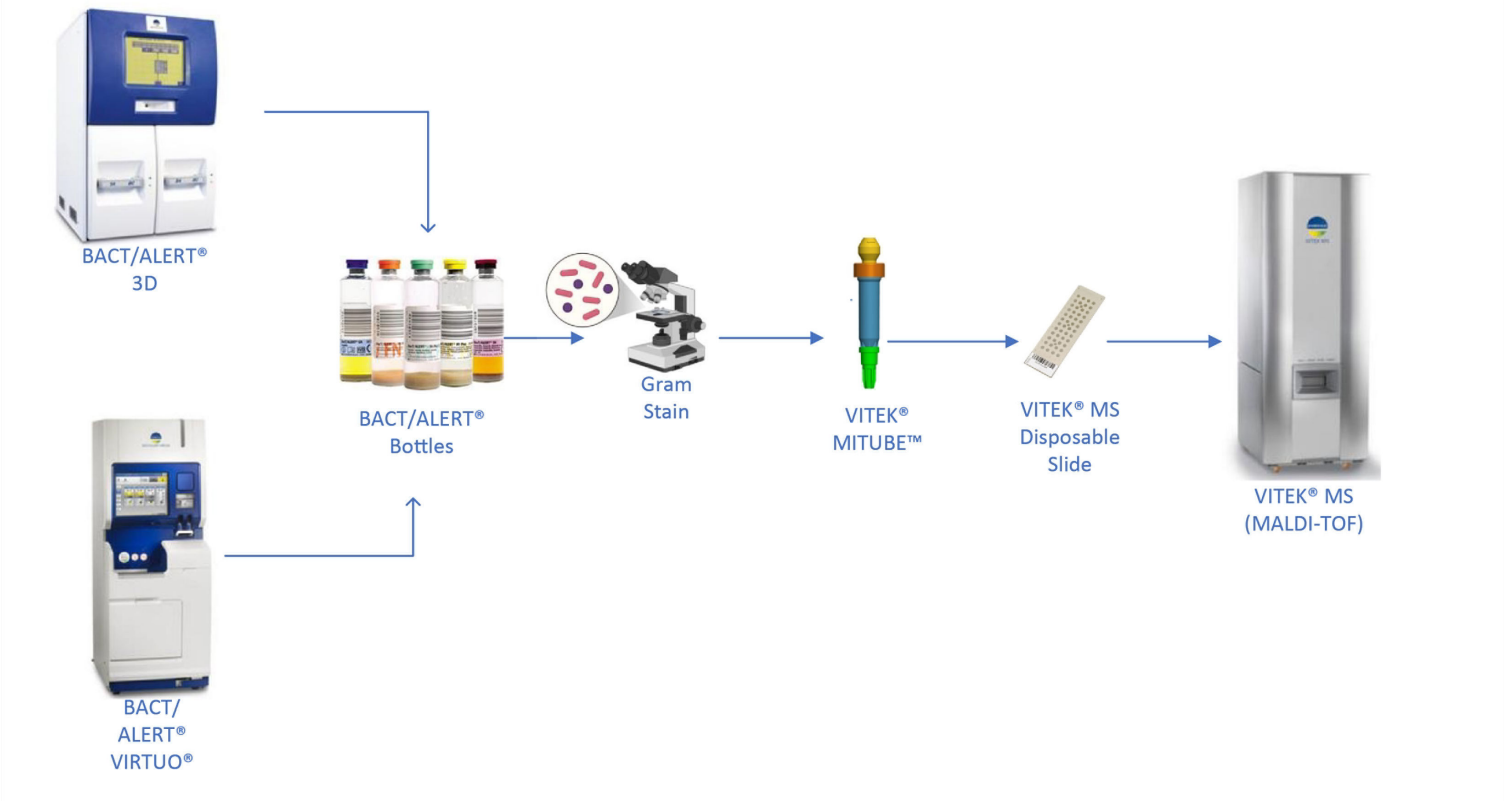
Sample flow for VITEK MITUBE testing in the prospective clinical study. The sample workflow from positive blood culture bottle to MALDI-TOF mass spectrometric identification is depicted. Once a blood culture bottle signals positive by an automated blood culture monitoring system, then the positive blood culture broth is removed. A Gram stain is performed, and cultures that appear to be monomicrobial gram-negative bacilli are carried forward to VITEK MITUBE for microbial clean-up, which results in a saline suspension of bacteria (see Fig. 2 for more details). The suspension is used to inoculate a MALDI target, which is then tested to identify the gram-negative bacteria.

Prospective clinical samples and contrived samples were used for the study. Three clinical sites obtained local institutional review board approval before enrolling clinical samples into the study. Subject-level data, including gender/sex, age, and hospitalization status, were not collected. Remnant PBC from BACT/ALERT 3D (bioMerieux) or BACT/ALERT VIRTUO (bioMerieux) was used in the study, and Fig. 1 depicts the sample workflow for the clinical samples.

For contrived blood culture samples, 10 mL of anticoagulated (sodium polyanethole sulfonate) whole blood was added to a bottle in addition to approximately 50 colony-forming units of bacteria. Different isolates of the same species were used for contrived testing, so the same isolate was not tested repeatedly. Bottles were then incubated on BACT/ALERT 3D or BACT/ALERT VIRTUO.

Blood culture bottles used in the study included BACT/ALERT FA Plus, BACT/ALERT FN Plus, BACT/ALERT PF Plus, BACT/ALERT SA, and BACT/ALERT SN. Positive BACT/ALERT bottles were intended to be tested promptly using the VITEK MITUBE to MALDI workflow and were not tested if more than 16 hours elapsed after bottle positivity was determined by BACT/ALERT 3D or VIRTUO. Before MITUBE processing, a Gram stain and subculture were performed. Samples with mixed morphologies identified by Gram stain were not tested in the study as the MITUBE device is only intended for use with monomicrobial samples.

Exclusion criteria included samples that could not be appropriately interpreted, samples that included isolates that were not claimed taxa (Box 1), contrived samples that were associated with a negative control blood bottle with growth due to contamination, samples associated with an unacceptable QC result on the day of testing, blood culture bottles that were processed outside of the defined timeframe, samples with incomplete or inadequate testing or documentation thereof, and duplicates or clones of the same organism isolated from the same subject.

Box 1. **Organisms compatible with VITEK MITUBE identification and attempted to be prospectively enrolled in the current study**

*Acinetobacter baumannii* *

*Klebsiella aerogenes*

*Enterobacter cloacae* spp. *cloacae* *

*Enterobacter cloacae* spp. *dissolvens* *

*Escherichia coli*

*Klebsiella oxytoca*

*Klebsiella pneumoniae*

*Proteus mirabilis*

*Proteus vulgaris* *

*Pseudomonas aeruginosa*

*Serratia marcescens*

*Acinetobacter baumannii, *Enterobacter cloacae*, and *Proteus vulgaris* were intended to be included in the study but were not encountered in the prospectively enrolled clinical samples.

**Fig 2:**
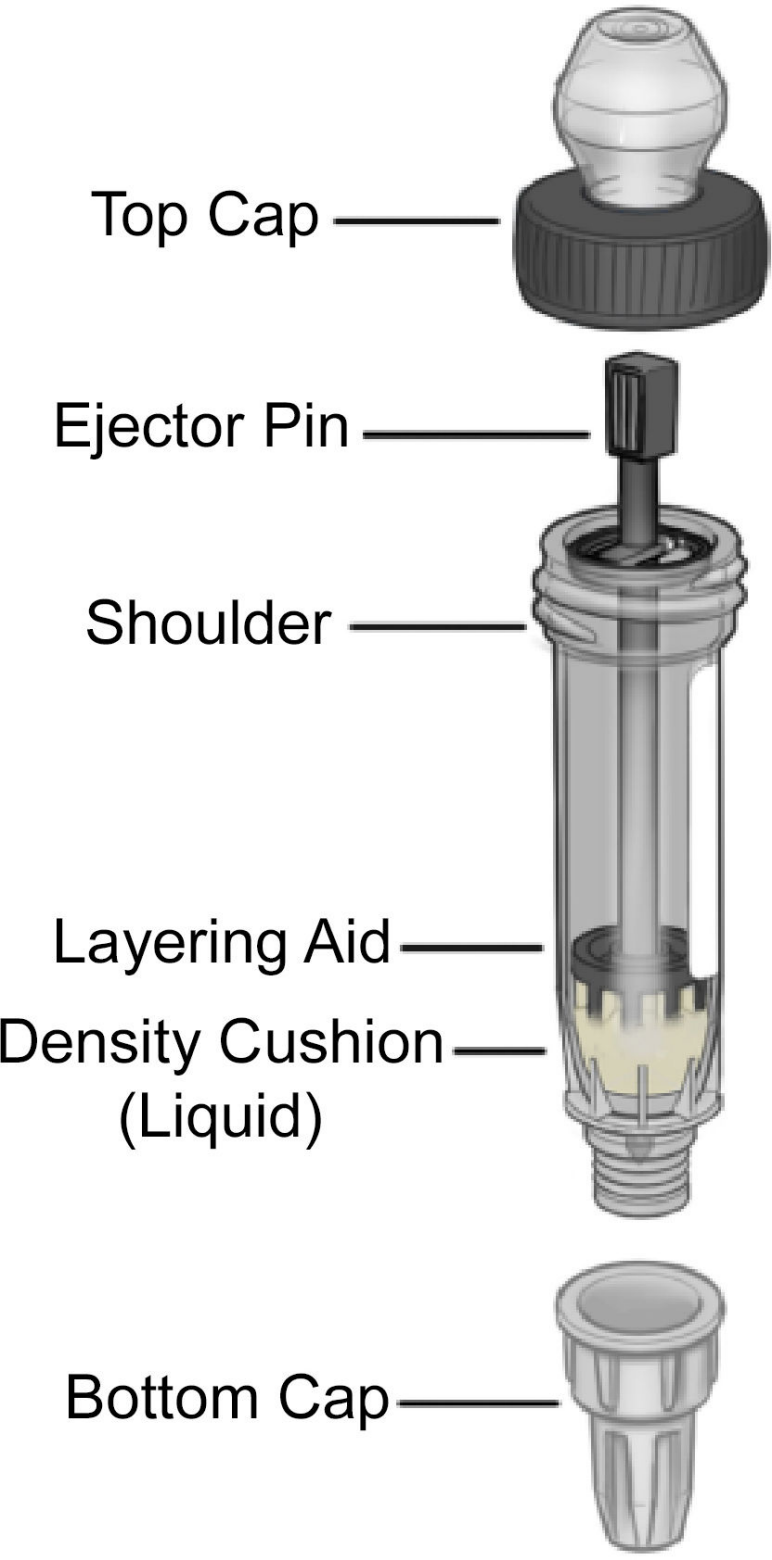
Exploded diagram of the VITEK MITUBE device. The components of the VITEK MITUBE device are labeled. The specimen processing workflow is as follows. After removing the top cap, the device is loaded with 6 mL of positive blood culture broth containing gram-negative bacteria that has been mixed 1:2 with lysis buffer. Then, the top lid is returned to the device, and it is centrifuged for 10 minutes. After centrifugation, the bottom cap is removed, the ejector pin is twisted, and the microbial pellet is dispensed into 1 mL of saline. Then, 1.0 µL of bacterial saline suspension is added to a pre-warmed VITEK MS target, followed by formic acid and then matrix. The bacterial isolate is then identified using VITEK MS.

Each positive bottle was inverted three to five times, and 2.5 mL of positive blood was removed aseptically with an 18-gauge needle and transferred into the lysis buffer tube. The lysis buffer tube was then vortexed at 2,500–3,000 rpm for 5 seconds twice. The design of the VITEK MITUBE device is depicted in Fig. 2. After the VITEK MITUBE device was centrifuged for approximately 5–10 seconds at 3,000 (× *g*) to ensure the density cushion was properly positioned, 6 mL of the lysate was transferred into the VITEK MITUBE device. The VITEK MITUBE device was then kept in an upright position and centrifuged for 10 minutes at 3,000 (× *g*). After visually confirming two distinct liquid layers were present in the device, the lower end cap of the VITEK MITUBE was removed, and the device was inserted into a polystyrene tube containing 1.0 mL of sterile saline. The ejector pin of the device was then twisted clockwise and fully depressed to introduce the pellet to the saline. The saline tube, with the VITEK MITUBE device still connected, was shaken to rinse the microbial pellet from the ejector pin. The saline tube and VITEK MITUBE were separated, and the saline tube was capped and vortexed for 3 seconds. Biomass in the saline tube was confirmed to be adequate if ≥0.50 McFarland was measured using the VITEK DENSICHEK. If the <0.50 McFarland was identified, then the sample was considered to have “low biomass” that was inadequate for testing. If the sample had adequate biomass, then a VITEK MS Slide was then prepared by spotting two different spots with 1.0 µL of suspension onto a MALDI target. The same spots received 0.5 µL of formic acid (VITEK MS-FA reagent, bioMérieux) and also 1.0 µL of VITEK MS-CHCA matrix (bioMérieux). The slides were then tested using the VITEK MS or VITEK MS PRIME instrument. A subset of the existing MALDI knowledge base was used, which included a limited number of taxa (Box 1).

If both spots were considered “No ID” (no identification) by MALDI, then the acquisition was repeated (the laser was “refired”) on both spots. If still receiving a No ID, then the initial suspension was reprocessed by washing; 100 µL was removed from the initial saline tube and placed into a 2 mL microcentrifuge tube. A volume of 0.9 mL of sterile saline was added to the tube, and the sample was vortexed for 5 seconds. The sample was then centrifuged at 14,000 (*× g*) for 2 minutes. The supernatant was removed with a pipette while preserving the pellet. The pellet was then resuspended into 50 µL of sterile deionized water. Two new deposits were prepared on the VITEK MS slide and covered with VITEK MS-FA and VITEK MS-CHCA Matrix. Acquisition was then attempted.

Reference method identification of the isolates was determined using the VITEK MS following the instructions for use, which require using an isolated colony grown on agar for testing. Only isolates with IVDR CE mark in Europe are described in this study (Box 1). Other microbes were excluded in this report.

The PBC VITEK MITUBE identification workflow also used VITEK MS or MS PRIME for identification, but the sample was prepared from the PBC instead of from an isolated colony. Initially, VITEK MS KB 3.2 was used, but the database was updated to KB 3.3 during the study. All spectra were reanalyzed using KB 3.3, and only data from KB 3.3 are included in this report; isolates initially identified using KB 3.2 but not identified or interpretable with the reference method using KB 3.3 were excluded from this analysis. Isolates were also excluded if the available spectra acquired with KB 3.2 did not reflect the repeat testing procedure when needed with KB 3.3.

## RESULTS

One hundred forty-seven (147) gram-negative prospective samples were considered for the study. Of these 147, 11 were excluded due to low biomass. Of the remaining 136, only 129 were taxa that can be identified by the test system (Box 1). One hundred twenty-five (125) of these 129 were included in the final data set because 4 isolates were excluded. These four isolates were excluded because the available spectra did not allow complete reanalysis with KB3.3, i.e., either there were no available spectra for the repeat testing procedure as it was not necessary with KB3.2 (one isolate) or the reference method results were no longer interpretable with KB3.3 (no identification results for two isolates and low discrimination results for one isolate). These four isolates include one *E. coli* isolate and three *Enterobacter cloacae*/*asburiae* isolates. Three of these isolates were accurately identified using MITUBE with KB 3.2, and one of the *Enterobacter* species did not achieve identification using the MITUBE workflow and KB 3.2. None of these four isolates were misidentified using KB 3.2. Samples that deviated from the protocol (e.g., samples that were determined to be polymicrobial, isolates with no definitive reference identification, samples processed outside of the timeframes described in the Materials and Methods, and samples with incomplete testing or incomplete documentation of testing) were excluded and not considered in the analysis.

Table 1 describes the performance for the 125 prospective PBC clinical samples included in the final data set, and each sample was tested at 1 of 3 clinical laboratory sites in the U.S. These taxa were encountered: *Escherichia coli* (69), *Klebsiella aerogenes* (4), *Klebsiella oxytoca* (3), *Klebsiella pneumoniae* (29), *Proteus mirabilis* (9), *Pseudomonas aeruginosa* (10), and *Serratia marcescens* (1). The large majority (98.4%) were accurately identified using the MITUBE to MALDI workflow. None were misidentified, and two (1.6%) *K. pneumoniae* were not identified.

**TABLE 1:** Performance of the VITEK MITUBE identification compared to the reference standard identification for prospectively enrolled clinical samples* of positive blood culture broth

Organism identification using reference method^*a*^	Quantity of isolates	Organism identification using the VITEK MITUBE system		
		No identification obtained	Incorrect identification obtained	Accurate species-level identification obtained
*Escherichia coli*	69	–^*b*^	–	69 (100%)
*Klebsiella aerogenes*	4	–	–	4 (100%)
*Klebsiella oxytoca*	3	–	–	3 (100%)
*Klebsiella pneumoniae*	29	2 (7%)	–	27 (93%)
*Proteus mirabilis*	9	–	–	9 (100%)
*Pseudomonas aeruginosa*	10	–	–	10 (100%)
*Serratia marcescens*	1	–	–	1 (100%)
Total	125	2 (1.6%)	0 (0%)	123 (98.4%)

^*a*^ *Acinetobacter baumannii*, *Enterobacter cloacae*, and *Proteus vulgaris* were not encountered in the prospectively enrolled clinical samples.

^*b*^ “–” indicates none.

Table 2 describes the combined performance of both the prospective PBC clinical samples described in Table 1 together with the contrived PBC samples described in Table 3. Accurate species-level identification was obtained in 95% (343/360) of the tests, which included accurate identifications for 95% (325/342) of the isolates. No identification was obtained in 4.4% (16/360) of the tests, and most of these (13/16) were due to *P. vulgaris*, which was only successfully identified in about half (18/31) of the attempts. One misidentification occurred in which *P. mirabilis* was misidentified as *E. faecalis*.

**TABLE 2:** Performance of the VITEK MITUBE identification compared to the reference standard identification for prospectively enrolled clinical samples of positive blood culture broth and contrived blood culture broth samples

Organism identification using reference method	Quantity of isolates	Organism identification using the VITEK MITUBE system		
		No identification obtained	Incorrect identification obtained	Accurate species-level identification obtained
*Acinetobacter baumannii^a^*	25	–^*e*^	–	25 (100%)
*Enterobacter cloacae* ssp. *cloacae^a^*	25	–	–	43 (100%)^*b*^
*Enterobacter cloacae* ssp. *dissolvens^a^*	2	–	–	2 (100%)
*Escherichia coli*	75	–	–	75 (100%)
*Klebsiella aerogenes*	28	–	–	28 (100%)
*Klebsiella oxytoca*	28	–	–	28 (100%)
*Klebsiella pneumoniae*	38	2 (5.3%)	–	36 (94.7%)
*Proteus mirabilis*	31	1 (3.2%)	1 (3.2%)^*c*^	29 (93.5%)
*Proteus vulgaris^a^*	31	13 (41.9%)	–	18 (58.1%)
*Pseudomonas aeruginosa*	33	–	–	33 (100%)
*Serratia marcescens*	26	–	–	26 (100%)
Total^*d*^	342	16 (4.4%)	1 (0.3%)	343 (95.3%)

^*a*^ *Acinetobacter baumannii*, *Enterobacter cloacae*, and *Proteus vulgaris* were not encountered in the prospectively enrolled clinical samples.

^*b*^ Eighteen (18) of the *Enterobacter cloacae* ssp. *cloacae* isolates were tested on both VITEK MS and VITEK MS PRIME and thus have two test results for one isolate.

^*c*^ The incorrect identification was obtained when analyzed by KB 3.3. The VITEK MITUBE workflow provided *Enterococcus faecalis,* whereas the isolated colony workflow provided *Proteus mirabilis*. Sequencing identification confirmed *P. mirabilis*.

^*d*^ 342 specimens were tested leading to 360 test results^*b*^. Among them, 125 were prospective clinical specimens (described in Table 1) and 217 were contrived specimens (described in Table 3).

^*e*^ “–” indicates none.

**TABLE 3:** Performance of the VITEK MITUBE identification compared to the reference standard identification for contrived blood culture broth samples

Organism identification using reference method	Quantity of isolates	Organism identification using the VITEK MITUBE system		
		No identification obtained	Incorrect identification obtained	Accurate species-level identification obtained
*Acinetobacter baumannii*	25	–^*d*^	–	25
*Enterobacter cloacae* ssp. *cloacae*	25	–	–	43^*a*^
*Enterobacter cloacae* ssp. *dissolvens*	2	–	–	2
*Escherichia coli*	6	–	–	6
*Klebsiella aerogenes*	24	–	–	24
*Klebsiella oxytoca*	25	–	–	25
*Klebsiella pneumoniae*	9	–	–	9
*Proteus mirabilis*	22	1 (4.5%)	1 (4.5%)^*b*^	20 (90.9%)
*Proteus vulgaris*	31	13 (41.9%)	–	18 (58.1%)
*Pseudomonas aeruginosa*	23	–	–	23
*Serratia marcescens*	25	–	–	25
Total^*c*^	217	14 (6.0%)	1 (0.4%)	220 (93.6%)

^*a*^ Eighteen (18) of the *Enterobacter cloacae* ssp. *cloacae* isolates were tested on both VITEK MS and VITEK MS PRIME and thus have two test results for one isolate.

^*b*^ The incorrect identification was obtained when analyzed by KB 3.3. The VITEK MITUBE workflow provided *Enterococcus faecalis,* whereas the isolated colony workflow provided *Proteus mirabilis*. Sequencing identification confirmed *P. mirabilis*.

^*c*^ 217 specimens were tested leading to 235 test results^*a*^.

^*d*^ “–” indicates none.

Table 4 describes the isolates that were acquired with initial testing and acquired after attempted reacquisition of spectra by refiring on the same target spots. No identifications were obtained by washing the suspension and attempting acquisition from newly created target spots.

**TABLE 4:** Performance of the MITUBE to MALDI workflow in achieving identification of on-label^*a*^ gram-negative bacteria in 125 prospective positive blood culture clinical samples across 3 laboratory sites

Identified with initial attempt	Identified after refiring the laser	Not identified	Misidentified
121 (96.8%)	2 (1.6%)	2 (1.6%)	0 (0%)

^*a*^ See Box 1 for on-label taxa.

## DISCUSSION

The VITEK MITUBE to MALDI workflow produced accurate identifications of bacteria directly from PBC without the need to subculture to solid media or use a molecular identification system. Initial MALDI acquisition identified 96.8% (121/125) of prospective isolates, and repeat firing on the same target spot increased the identification frequency to 98.4% (123/125). Washing the bacterial suspension and making new target spots did not yield identifications for the two isolates that were not initially identified.

Some of the taxa intended to be included in the study were not encountered in the prospective clinical PBC samples (Table 1), and the taxa encountered infrequently or not at all during prospective enrollment were assessed using contrived samples, which demonstrated similar performance with the exception of *P. vulgaris*, which frequently was not able to be identified using the VITEK MITUBE to MALDI workflow (Table 2) (4).

Over the past decade, many clinical microbiology laboratories have implemented direct-from-PBC molecular testing to rapidly identify the organism present in the PBC and, in some cases, to characterize the genotypic antimicrobial resistance determinants (5, 6). In the coming decade, we anticipate clinical microbiology laboratories will more widely adopt direct from PBC systems for rapid antibiotic susceptibility testing (AST) as more commercial AST systems achieve regulatory clearance for directly testing PBC. These rapid AST systems will likely be paired with rapid identification systems, and it is not yet clear if clinical laboratories will continue to use molecular panels for rapid identification from PBC or if labs will transition to fast MALDI solutions for identification, such as SepsiTyper (Bruker) or VITEK MITUBE, or to other methods, such as Accelerate Arc. We anticipate the decision to maintain molecular vs transition to MALDI for PBC identification will be influenced by multiple variables, including the rapidity, affordability, simplicity, detection of resistance genes, and accuracy of the MALDI solutions as well as the perceived need to implement rapid AST, which would need to be paired with a rapid identification system. The VITEK MITUBE system has a benefit in that the system could be employed with minimal capital expenditure and little or no new equipment acquisition for laboratories that currently use VITEK MS or MS PRIME.

The VITEK MITUBE system is not marketed in the U.S., and its IVDR CE mark in Europe is limited to frequently encountered gram-negative bacteria (Box 1). Expanding the indications for use to include more taxa could enhance the utility of the system.

This clinical study evaluated the performance of VITEK MITUBE to aid in the fast identification of gram-negative bacterial isolates directly from PBC, and the performance characteristics suggest that VITEK MITUBE could be a good solution for clinical laboratories that are working to expedite identification of gram-negative blood culture isolates.
